# The prevalence of *Dirofilaria repens* in cats, healthy dogs and dogs with concurrent babesiosis in an expansion zone in central Europe

**DOI:** 10.1186/s12917-016-0816-3

**Published:** 2016-09-05

**Authors:** Anna Bajer, Anna Rodo, Ewa J. Mierzejewska, Katarzyna Tołkacz, Renata Welc-Faleciak

**Affiliations:** 1 Department of Parasitology, Institute of Zoology, Faculty of Biology, University of Warsaw, 1 Miecznikowa Street, 02-096 Warsaw, Poland; 2 Department of Pathology and Veterinary Diagnostics, Warsaw University of Life Sciences- SGGW, 159c Nowoursynowska Street, 02-766 Warsaw, Poland; 3 Lab-Wet, Veterinary Diagnostic Laboratory, ul. Wita Stwosza 30, 02-661 Warsaw, Poland

**Keywords:** *Dirofilaria repens*, *Babesia canis*, Co-infection, Dogs, Cats, Thrombocytopenia, Hepatic failure, Renal failure, Creatinine, Serum urea

## Abstract

**Background:**

*Dirofilaria repens* is a mosquito-transmitted, filarial nematode parasitizing dogs, cats and other carnivores. Recently, this parasite has spread in central Europe, including Poland. The aim of the present study was to estimate the prevalence of *D. repens* in cats and dogs in different regions of the country and to investigate the occurrence and consequences of co-infection with another fast-spreading vector-borne parasite, *Babesia canis*.

**Results:**

In the period 2013–2015, 147 blood samples from cats from central Poland and 257 blood samples from dogs from central, northern, southern and western Poland were collected. Prevalence of *D. repens* was determined by amplification and sequencing of the *12S rDNA* gene fragment. Among dogs, 94 samples originated from clinically healthy dogs from central Poland (Masovia) and 58 samples originated from dogs that were infected with *B. canis*. Prevalence of *D. repens* was compared between these two groups of dogs.

For the first time *D. repens* was identified in a cat from central Europe (0.7 % [95 % CL: 0–4.1 %]). The DNA of the filarial endosymbiotic bacterium *Wolbachia* was detected in two cats (1.4 % [95 % CL: 0–5.5 %]). In dogs, the parasite was detected only in samples from central Poland (Masovia) (local prevalence = 38 % [95 % CL: 25.9–51.8 %]). Prevalence of *D. repens* was significantly higher in dogs with babesiosis (90 % [95 % CL: 81.6–94.5 %]). Co-infections of *D. repens* and *B. canis* were confirmed by sequencing in 30 dogs with babesiosis, but no co-infections were identified in healthy dogs from Masovia. Statistical analyses of blood parameters revealed that dogs with co-infections suffered more severe anemia and thrombocytopenia, but presented milder changes in biochemical parameters (i.e. less elevated concentration of alkaline phosphatase [ALP] and serum urea) suggesting lower risk of hepatic or renal failure in comparison to dogs infected only with *B. canis*.

**Conclusions:**

These findings are important due to the spread of dirofilariosis and babesiosis in central Europe, as microfilaraemic dogs seem to be more prone to babesiosis. The possible protective effect of the nematode infection against hepatic or renal failure in canine babesiosis and its mechanisms require further investigations.

## Background

*Dirofilaria repens* is a filarial species transmitted by more than 60 mosquito species acting as intermediate hosts of the parasite [1]. Dogs and other carnivores are its final hosts and humans are incidental hosts. *Dirofilaria repens* is a well-recognized parasite of dogs and humans in southern Europe [1–4] in the Ukraine [5, 6] and southern Russia [7]. Although cats appear to be competent hosts for *D. repens*, they are much more resistant to invasion and in endemic regions prevalence is considerably lower than in dogs [2].

Recently, the emergence of canine and human dirofilariosis due to *D. repens* has been reported in central European countries, a region that was previously considered to be non-endemic for this parasite species [2, 8–14]. Among the most probable reasons for this northward expansion of *D. repens* in Europe are climatic changes associated with global warming (facilitating the development of larval stages in the mosquito host) and translocation of infected dogs from southern to central Europe [15, 16]. The total number of human cases of dirofilariosis has changed dynamically, increasing from 1200 cases reported in 2009 [2] to over 4000 cases reported in 2015 [17], an increasing statistic that is likely also to be mainly attributable to a greater interest in the epidemiology of dirofilariosis in recent years. Thus dirofilariosis caused by *D. repens* is now considered to be an emerging zoonosis in Europe [1].

Dogs serve as the major source of infection of *D. repens* for mosquitoes which then transmit infection to humans. Therefore, the recognition, treatment and prevention of *D. repens* invasion in dogs is likely to help also the control of infection in humans [2]. Although there are new measures for the elimination of adult *D. repens* worms from dogs [18], a key problem is that neither the diagnosis nor the implementation of preventative/ control measures are common in newly established endemic regions in central Europe. Recent studies on the distribution of *D. repens* in Slovakia and Poland have detected high prevalence (34–60 %) of infection in clinically healthy working dogs [8, 19] and these asymptomatic infections are not usually recognized and treated. The presence of adult *D. repens* in dogs causes canine subcutaneous dirofilariosis, with a range of dermatological manifestations [20], but apparently may also be asymptomatic and chronic for several years [2, 8, 19]. The pathogenicity associated with accumulating high numbers of microfilariae in the circulating blood of infected dogs is also not well recognized but there are a few reports on possible acute and fatal outcomes of such infections associated with the presence of microfilariae in vital organs (heart, liver, kidney) [21].

*Dirofilaria repens* infections in dogs, humans and vectors have been reported often in recent years in countries in the vicinity of Poland [6, 13, 15, 17, 19]. In Germany at least two endemic regions have been recognized: between Stuttgart and Frankfurt/Men and in Branderburg federal state (near Berlin and possibly in the Oder River valley) [13, 15, 22, 23] In Slovakia, *D. repens* in dogs was recorded for the first time in 2005, and to date is widely spread and has been detected in *Aedes vexans* mosquitoes [11, 12, 19, 24]. In the Ukraine, dirofilariosis is a registerable zoonotic disease and about 91 new cases in humans are registered each year [6, 17].

To our knowledge, no data on the prevalence of *D. repens* in cats in central Europe is available.

In Poland, *D. repens* was recorded for the first time in a dog in 2009 [25] but already Central Poland is now well recognized as a recently established endemic region for this species, with a high prevalence having been reported in dogs, then in mosquitoes and as autochthonous zoonotic invasions in humans [5, 14, 26]. However, the distribution of the parasite in other regions of Poland is less well recognized.

The high prevalence of *D. repens* infections in dogs/ cats constitutes a new challenge for veterinarians due to the increasing probability of co-infection occurring with other blood parasites vectored by ticks [19]. Among the vector-borne diseases of greatest importance is canine babesiosis, caused by *Babesia canis* transmitted by *Dermacentor reticulatus* ticks [27–29]. In central and eastern regions of Poland canine babesiosis is endemic and hyper-endemic, causing thousands of life-threatening infections in dogs each year. As an example, over a period of 6 months (autumn 2013 to spring 2014) over 1200 canine babesiosis cases were treated in selected vet clinics in two districts in eastern Poland (Wysokie Mazowieckie and Semiatycze, Podlaskie voivodeship [large administrative region]) [30]. Prevalence of *B. canis* infections in *D. reticulatus* ticks may be as high as 8–16 % in central Poland [28] and this tick species dominates on dogs in this part of Poland [31]. To our knowledge, the impact of concurrent infections of *D. repens* with *B. canis* has not been investigated. Therefore we assessed the prevalence of *D. repens* and its effect on the host in dogs infected with *B. canis*, originating from the Mazovia region.

The aims of the present work were: (1) to estimate the prevalence of *D. repens* infection in cats and dogs from different regions of Poland (2) to compare the prevalence of *D. repens* in healthy dogs and dogs with babesiosis and finally (3) to analyze the influence of *D. repens* infections on the severity of canine babesiosis.

## Methods

### Sampling

Overall, 147 blood samples from cats were included in this study. These originated from pet cats that were regularly allowed to roam freely outside the house or apartment of their owners, and were provided by the Lab-Wet diagnostic laboratory in the period 2013–2014.

In total, 257 individual blood samples were collected from dogs between October 2014 and November 2015, including samples from healthy dogs from central Poland, Warsaw area (*n* = 94; diverse group of dogs- pet dogs, sled dogs, dogs from shelters), samples from dogs from northern Poland (Gdańsk *n* = 34; Bydgoszcz *n* = 5); samples from southern Poland (Kraków *n* = 30; Katowice *n* = 6) and samples from western Poland (Wrocław *n* = 30). Samples from Gdańsk, Kraków and Wrocław were provided by Lab-Wet diagnostics laboratory, and originated from dogs referred for blood analysis because of a range of different medical reasons.

In addition to these 199 samples, the occurrence of *D. repens* was investigated among 58 dogs suspected for babesiosis. These samples originated from dogs presenting with a wide range of babesiosis symptoms, including fatal cases, originating from central Poland in the vicinity of Warsaw, a region that is known to be endemic both for canine babesiosis and dirofilariosis. Samples were enrolled into the study on the basis of a positive diagnosis of *B. canis* infection as detected by the Lab-Vet diagnostics laboratory.

To study the effect of concurrent infection with *D. repens* and *B. canis*, we analyzed standard data on blood cell concentrations (and related counts) available from 41 dogs and biochemistry parameters available from 37 dogs suffering from babesiosis. Blood parameters were compared between dogs with single (*B. canis*) and double (*B. canis* + *D. repens*) infections.

### Molecular analysis

Infections with *D. repens* and *B. canis* in dogs were identified/confirmed based on specific PCR amplification and sequencing of reference genes [32–34]. Additionally, to detect *D. repens* invasions in cats, a fragment of the *ftsZ* gene of the filarial endosymbiotic bacterium, *Wolbachia*, was amplified and sequenced following Turba et al. (2012) [35]. Detection of *Wolbachia* in blood samples constitutes an indirect, but sensitive method for the assessment of the presence/absence of filarial infections.

Blood samples were collected into 0.001 M EDTA and frozen at a temperature of -20 °C until DNA extraction. DNA extractions were performed on whole blood using the AxyGen MiniPrep Blood kit (AxyGen, USA) or MO BIO Ultra Clean Blood Spin DNA Isolation kit (MO BIO Laboratories, USA). A combination of *D. repens* specific primers (12SF and 12SR2) extracted from a multiplex PCR developed by Gioia et al. (2010) [34] was used to amplify the *12S rRNA* gene fragment (327 bp) of *D. repens* [8]. Amplification of the *18S rRNA Babesia* gene fragment was performed using the previously described PCR protocol [32, 33]. Primers BAB GF2 (5′ GYYTTGTAA TTGGAATGATGG 3′) and BABGR2 (5′ CCAAAGACTTTGATTTCTCTC 3′) were used to produce a ~550 bp fragment. Positive controls were as follows: for *Babesia* detection*- B. microti* King’s College strain genomic DNA [36]; for *D. repens- D. repens* genomic DNA from an adult nematode kindly provided by Dr. Aleksander Masny, National Institute of Health in Warsaw. Positive and negative (sterile H_2_O as template) controls were included in each set of PCRs.

Amplicons were visualized with Midori Green stain (Nippon Genetics Europe GmbH, Germany) following the electrophoresis in 1.5 % agarose gels. Selected amplicons were purified and sequenced by a private company (Genomed S.A., Poland). DNA sequence alignments were conducted using MEGA version 6.0 [37]. The resulting sequences were compared with sequences deposited in GenBank NCBI.

### Statistical methods

Prevalence of *D. repens* infection (% infected) in dogs with and without babesiosis was compared by Fisher exact test, INSTAT software. Comparison of mean blood parameter values between dogs with single (*B. canis*) and double (*B. canis + D. repens*) infections was carried out by Student’s t-tests, implemented by the software package, IBM SPSS v. 21 (Table 1). An ANOVA was also used for the comparison of mean blood parameter values between dogs categorized in 3 classes of severity of babesiosis- to confirm correct categorization of scores (Table 2). The presence/absence of *D. repens* infection, fitted as a binary factor (infected =1, not infected =0) was included in a second ANOVA, incorporating also severity scores of babesiosis or scores for the severity of appropriate ‘general’ symptom as the dependent variables (anemia, hepatic or renal dysfunction, see below) (Table 3). These analyses were carried out to test our research hypothesis, that severity of babesiosis may be affected by concurrent *D. repens* infection through the negative impact of microfilariae on a range of blood parameters and the function of vital organs (liver, kidney) (as indicated in [19]). Thus, we performed a series of statistical comparisons with the presence/absence of *D. repens* as a factor associated with severity scores for three main ‘general’ symptoms and an overall severity score for babesiosis (explained below).

**Table 1:** Comparison of blood parameters between dogs co-infected with *B. canis* and *D. repens* and infected only with *B. canis*

Parameter	Mean parameter value ± SEM			Reference values	*P*
	Overall	Dogs with co-infection	Dogs with *B.canis*		
Counts (*n* = 41):		*n* = 36	*n* = 5		
Leukocytes (G/l)	5.850 ± 0.551	5.039 ± 0.320	7.50 ± 2.914	6.0–12.0	0.007
Erythrocytes (T/l)	4.885 ± 0.115	4.976 ± 0.191	4.18 ± 0.401	5.5–8.0	0.758NS
Hemoglobin (mmol/l)	7.238 ± 0.180	7.161 ± 0.254	7.404 ± 0.868	7.45–11.17	0.747 NS
Hematocrit (l/l)	0.332 ± 0.008	0.326 ± 0.012	0.342 ± 0.038	0.37–0.55	0.660 NS
MCV (fl)	67.26 ± 0.944	66.11 ± 0.778	70.44 ± 2.737	60–77	0.068 NS
MCHC (mmol/l)	21.86 ± 0.147	21.97 ± 0.109	21.58 ± 0.426	19.8–22.3	0.246 NS
Thrombocytes (G/l)	57.09 ± 5.007	45.40 ± 3.335	91.80 ± 26.79	200–580	0.001
Biochemical parameters (*n* = 37):		*n* = 32	*n* = 5		
AST (U/l)	120.66 ± 31.58	113.28 ± 18.83	249.40 ± 159.07	1–45	0.079 NS
ALT (U/l)	139.93 ± 39.63	126.25 ± 33.84	312.40 ± 156.41	3–60	0.081 NS
ALP (U/l)	203.98 ± 39.56	165.48 ± 20.71	432.80 ± 206.45	20–155	0.007
Serum glucose (mg/dl)	105.43 ± 7.066	100.33 ± 4.965	125.00 ± 30.33	70–120	0.151 NS
Creatinine (mg/dl)	1.541 ± 0.121	1.125 ± 0.134	1.780 ± 1.031	0.8–1.7	0.206 NS
Serum urea (mg/dl)	108.96 ± 8.376	61.50 ± 14.26	135.20 ± 98.20	20–45	0.158 NS
Total serum protein (g/l)	60.01 ± 1.177	59.59 ± 1.112	59.20 ± 2.437	55–75	0.896 NS

*SEM* standard error of the mean, *P* probability values from Student’s t-tests, *MCV* mean corpuscular volume, *MCHC* mean corpuscular hemoglobin concentration, *AST* aspartate aminotransferase, *ALT* alanine aminotransferase, *ALP* alkaline phosphatase

**Table 2:** Comparison of the selected parameters for the estimation of the severity of the three general symptoms, then used for classification of the severity of the babesiosis (classes 1–3)

Summarized symptom and parameters ± SEM	Severity class			*P*
	Class 0 (normal)	Class 1 (mild)	Class 2 (severe)	
1. Anemia:	*n* = 15	*n* = 14	*n* = 12	
Erythrocytes (T/l)	5.98 ± 0.183	5.02 ± 0.187	3.20 ± 0.235	0.000
Leukocytes (G/l)	4.89 ± 0.874	4.69 ± 0.896	9.06 ± 1.126	0.014
Hematocrit (l/l)	0.402 ± 0.013	0.347 ± 0.013	0.213 ± 0.017	0.000
Hemoglobin (mmol/l)	8.777 ± 0.286	7.558 ± 0.293	4.651 ± 0.368	0.000
Thrombocytes (G/l)	45.17 ± 7.945	59.83 ± 8.143	68.43 ± 10.234	0.103 NS
2. Hepatic dysfunction:	*n* = 5	*n* = 22	*n* = 10	
AST (U/l)	32.13 ± 77.238	78.20 ± 43.727	315.33 ± 47.672	0.029
ALT (U/l)	37.25 ± 96.948	66.58 ± 54.886	426.00 ± 59.838	0.011
ALP (U/l)	112.50 ± 96.599	141.69 ± 54.840	451.19 ± 59.622	0.026
3. Renal dysfunction:	*n* = 25	*n* = 7	*n* = 5	
Creatinine (mg/dl)	0.802 ± 0.151	0.983 ± 0.240	4.23 ± 0.304	0.000
Serum urea (mg/dl)	29.27 ± 10.459	55.92 ± 16.656	387.75 ± 21.116	0.000

*P-*probability values from ANOVA tests

Reference values for parameters and explanations as in legend to Table 1

**Table 3:** Comparison of blood parameters between dogs co-infected with *B. canis* and *D. repens* and infected only with *B. canis* in different severity classes

General symptom level	Parameter	Mean (± SEM) in dogs infected with *B.canis + D.repens*	Mean (± SEM) in dogs infected only with *B. canis*	F and *P*
I. Anemia				
Level 0:	Erythrocytes (T/l)	6.07 ± 0.181	6.00 ± 0.610	F _2, 40 _= 0.708; *P* = 0.408
	Leucocytes (G/l)	5.12 ± 0.756	4.20 ± 2.661	F _2, 40 _= 2.734; *P* = 0.082
	Thrombocytes (G/l)	52.56 ± 6.871	23.00 ± 24.188	F _2, 40 _= 6.866; *P* = 0.004
	Hematocrit (l/l)	0.377 ± 0.014	0.450 ± 0.046	F _2, 40 _= 0.044; *P* = 0.835
Level 1:	Erythrocytes (T/l)	4.80 ± 0.294	5.08 ± 0.431	
	Leucocytes (G/l)	4.39 ± 0.810	5.15 ± 1.881	
	Thrombocytes(G/l)	38.06 ± 7.360	92.50 ± 17.103	
	Hematocrit(l/l)	0.311 ± 0.022	0.365 ± 0.033	
Level 2:	Erythrocytes (T/l)	3.64 ± 0.249	3.95 ± 0.431	
	Leucocytes (G/l)	7.83 ± 1.402	11.50 ± 1.881	
	Thrombocytes (G/l)	39.89 ± 12.748	125.50 ± 17.103	
	Hematocrit (l/l)	0.247 ± 0.019	0.265 ± 0.033	
II. Hepatic dysfunction				
Level 0:	AST (U/l)	34.60 ± 24.153	nd	F _1, 36 _= 0.709; *P* = 0.407
	ALT (U/l)	40.40 ± 51.35	nd	F _1, 36 _= 0.044; *P* = 0.835
	ALP (U/l)	102.00 ± 48.093	nd	F _1, 36 _= 0.360; *P* = 0.554
Level 1:	AST (U/l)	93.78 ± 15.868	81.00 ± 38.189	
	ALT (U/l)	60.33 ± 33.736	82.00 ± 81.192	
	ALP (U/l)	151.28 ± 31.726	119.50 ± 76.041	
Level 2:	AST (U/l)	307.67 ± 23.815	361.67 ± 31.181	
	ALT (U/l)	378.08 ± 50.632	466.00 ± 66.293	
	ALP (U/l)	259.00 ± 47.420	641.67 ± 62.087	
III. Renal dysfunction				
Level 0:	Creatinine (mg/dl)	0.87 ± 0.117	0.70 ± 0.334	F _2, 36 _= 11.68; *P* = 0.000
	Serum urea (mg/dl)	28.94 ± 8.127	29.75 ± 23.131	F _2, 36 _= 16.16; *P* = 0.000
Level 1:	Creatinine (mg/dl)	1.03 ± 0.236	0.90 ± 0.545	
	Serum urea (mg/dl)	52.88 ± 16.356	62.00 ± 37.773	
Level 2:	Creatinine (mg/dl)	2.55 ± 0.272	5.90 ± 0.545	
	Serum urea (mg/dl)	248.50 ± 18.886	527.00 ± 37.773	

P values derived from ANOVAs

*D. repens* presence x anemia level on erythrocytes count; *D. repens* presence x anemia level on leucocytes count; *D. repens* presence x anemia level on thrombocytes count

*D. repens* presence x hepatic dysfunction level on AST activity; *D. repens* presence x hepatic dysfunction level on ALT activity; *D. repens* presence x hepatic dysfunction level on ALP activity; *D. repens* presence x renal dysfunction level on serum creatinine; *D. repens* presence x renal dysfunction level on serum urea

Nd- not done, the lack of cases in this group

The severity of babesiosis was classified at 3 levels. As all dogs with babesiosis (*n* = 58) suffered from thrombocytopenia (only two dogs had thrombocytes count in the range 100–150, all the others had counts below 100 G/l) (Table 1), this parameter was not included in the classification. The three main groups of general symptoms (indicators of mortality in canine babesiosis) included: a. severity of anemia (severity score 0- RBC counts within normal range 5.5–8.0 T/l; severity score 1- RBC counts in a range 4.5–5.5 T/l; severity score 2- RBC below 4.5 T/l); b. hepatic dysfunction parameters (measures of enzymes activity [based on reference values for AST – aspartate aminotransferase and ALT- alanine aminotransferase; with accompanying ALP – alkaline phosphatase] within, above (up to 100 U/l) and profoundly above (>100 U/l) the normal range, representing scores of 0, 1 and 2, respectively, Table 2); c. renal dysfunction parameters (based on reference values for serum urea within, above (up to 100 mg/dl) and profoundly above (>100 mg/dl) the normal range, representing severity scores of 0, 1 and 2, respectively, Table 2).

Then all dogs with any of these three general symptoms classified as ‘severity score 2’ were assigned to group 3 (severe babesiosis, severity level 3, *n* = 20); dogs presenting with more than one dysfunction (anemia, symptoms of hepatic or renal failure) at level 1 (abnormal) were assigned to group 2 (moderate babesiosis, severity level 2; *n* = 11) and finally dogs presenting only with one group of symptoms (mainly mild anemia and thrombocytopenia) were assigned to group 1 (mild babesiosis, severity level 1, *n* = 10). This final classification was carried out to assess the general impact of concurrent *D. repens* infection on the severity of babesiosis in dogs and appropriate levels were implemented into multifactorial ANOVAs for each dog for each blood parameter (Table 2).

Finally, log-linear analyses of contingency tables were performed (SPSS v. 21) to compare prevalence of *D. repens* in dogs assigned to three severity classes (4 different classification: 1. anemia [0–2]; 2. symptoms of hepatic failure [0–2]; 3. symptoms of renal failure [0–2]; babesiosis severity [1–3]). This analysis was carried out to test our research hypothesis that the prevalence of *D. repens* infection should be higher in dogs suffering more severe symptoms (our scores 2 and 3 for ‘general’ symptoms or babesiosis, respectively). Again, this was done to seek evidence for an association between severity of the disease and presence of *D. repens*. We tested two models: anemia scale x babesiosis scale x *D. repens* presence (0, 1) and hepatic dysfunction class x renal dysfunction class x babesiosis scale x *D. repens* prevalence (0, 1).

## Results

### The occurrence of *Dirofilaria repens* and *Wolbachia* in cats

#### Dirofilaria repens

DNA was detected only in one blood sample, from one cat positive also for *Wolbachia* (0.7 % [95 % CL: 0–4.1 %]). Our sequence was identical to the *D. repens* isolate obtained from the blood of a sled dog in our previous study (accession number KF494237; [8]). *Wolbachia* DNA was detected in 2 out of 147 cats (1.4 % [95 % CL: 0–5.5 %]). Both sequences were identical, displaying the highest homology (>99 %) to the sequence of *Wolbachia,* endosymbiont of *D. repens* (GenBank accession number AJ010273).

### The occurrence of *Dirofilaria repens* in dogs from different regions of Poland

#### Dirofilaria repens

DNA was detected only in dogs from central Poland. None of the samples from the other regions (southern, western or northern Poland) tested positive. The specific diagnostic 320 bp band of the *12S rRNA* gene fragment of *D. repens* was observed in 36 dogs out of 94 healthy dogs that were tested (prevalence 38.3 % [95 % CL: 25.9–51.8 %]). To confirm the specific amplification, 16 (44 % of all positive) randomly selected samples were sequenced, aligned and compared with sequences from the GenBank database. All sequences were identical, displaying the highest homology (295/ 296 identical nucleotides, 99.7 %) to the sequence of *D. repens* isolated from a subcutaneous human case in Russia (accession number KM205374). Our sequences displayed similar high homology (263/265 identical nucleotides, 99.2 %) with the *D. repens* isolate obtained from a sled dog in our previous study (accession number KF494237; [8]).

To test for the occurrence of possible unidentified, asymptomatic co-infections with *B. canis* and *D. repens*, we also analyzed samples from these 94 healthy dogs from central Poland for *Babesia* DNA. Only 1 asymptomatic dog (prevalence 1.06 % [95 % CL: 0–5.2 %]) proved positive for *B. canis* but it was not infected with *D. repens*.

### The occurrence of *Dirofilaria repens* in dogs with babesiosis in central Poland

First, to confirm the laboratory diagnosis of *B. canis* infection based on microscopical observation of blood smears, the 560 bp *18S rRNA* gene fragment of *Babesia* was amplified in blood samples from all symptomatic dogs (58/58 = 100 %). Then, the 320 bp *12S rRNA* gene fragment of *D. repens* was amplified with species-specific primers. *Dirofilaria repens* DNA was detected in 52 of 58 dogs with babesiosis (prevalence 89.7 % [95 % CL: 81.6–94.5 %]). The difference in prevalence of *D. repens* between healthy dogs and dogs with babesiosis (38.3 % versus 89.7 %) was highly significant (Fisher’s exact test, *P* < 0.0001).

To confirm the occurrence of co-infections of *B. canis* and *D. repens,* respective PCR products were sequenced from 30 double-positive dogs (57.7 % of all double-positive samples). Sequences were aligned and compared with the GenBank database. Sequencing revealed that 30 *Babesia* sequences displayed the highest homology (514/ 519 identical nucleotides; 99.0 %) to the *B. canis* genotype 2 (EU622793), originally isolated from a dog with babesiosis in Poland [38] and to a *B. canis* isolate from a *D. reticulatus* tick (KT272401; 516/520 = 99 %; [31]).

The sequencing of 30 *D. repens* positive amplicons revealed the presence of a *D. repens* variant identical with isolates obtained in this study from the healthy dogs from the same region of central Poland, displaying the highest homology (97–99 %) to the sequence of *D. repens* isolated from a human case in Russia (KM205374) and to a sequence from one of our dogs deposited earlier (KF494237). Lower homology for several sequences arose through some non-specific background amplification of canine DNA.

### The influence of *D. repens* infections on the severity of babesiosis

Various blood parameters were compared between two groups of dogs: 36 or 32 dogs co-infected with *B. canis* and *D. repens* and 5 dogs infected only with *B. canis* (Table 1).

Generally, mean numbers of erythrocytes, leucocytes, thrombocytes and mean values for hematocrit or hemoglobin concentration obtained for all 41 dogs with babesiosis were below reference values, but the majority of these values were lower in dogs with co-infection in comparison to dogs infected only with *B. canis*. However, these differences were significant only for the mean number of thrombocytes and leucocytes (Table 1). Thrombocyte numbers were twice as high in dogs with just *B. canis* compare to those with co-infection.

A contrasting pattern was observed for biochemical parameters. Although overall mean parameter values calculated for all dogs with babesiosis were higher relative to normal levels (values of AST, ALT and ALP and serum urea concentration), the means for dogs only infected with *B. canis* were higher than those of dogs with concurrent *D. repens* (Table 1), indicating a more severe babesiosis in the former dogs with involvement of hepatic and renal failure. Statistically significant differences were observed only for ALP activity (Table 1).

In the next step of analysis, we test our research hypothesis, that severity of babesiosis may be affected by concurrent *D. repens* infection through the negative impact of the presence of microfilariae on a range of blood parameters and the function of vital organs (liver, kidney). The symptoms were compiled to create three general classes of symptoms (anemia, symptoms of hepatic or renal failure; Table 2), each with three severity levels and implemented into statistical models (ANOVAs for specific parameter). Statistical analysis confirmed significant differences between the mean blood parameters of dogs arbitrarily assigned to three classes of severity (Table 2) and revealed some interesting effects of the interaction between the presence of *D. repens* and severity of general symptoms on mean values of selected parameters (Table 3). Contrary to our expectations, among the different parameters the values that were profoundly above normal level were observed in dogs assigned to class 2 and infected only with *B. canis*, and the range of means in three severity classes was generally lower in dogs with co-infections (i.e. differences in leucocytes, ALP, serum creatinine or urea concentrations between two groups of dogs in class 2; Table 3).

In the final step of our analysis, we examined associations between the prevalence of *D. repens* and the severity scores for babesiosis, anemia, symptoms of hepatic or renal dysfunction (between 3 respective classes). No statistically significant associations were obtained in these analyses. The only observed trend was for the association between the presence of *D. repens* and hepatic dysfunction classes (χ^2^ = 3.69, df = 2, *P* = 0.158). Prevalence of *D. repens* was highest in class 0 (normal) with all dogs co-infected with filariae (100 %); was 91 % in class 1 (abnormal) and the lowest (70 %) in dogs assigned to severity class 2 (hepatic failure), indicating an opposite association to that predicted in our research hypothesis.

## Discussion

The main aims of this study were to estimate the prevalence of *D. repens* in cats and dogs from different regions of Poland and to evaluate the influence of this nematode infection on concurrent canine babesiosis. We confirmed the high prevalence of *D. repens* infection in the recently established endemic region in central Poland and found no infected dogs outside this area. Interestingly, although the prevalence of *D. repens* was more than twice as high in dogs with babesiosis in comparison to healthy animals, statistical analyses of a range of blood parameters revealed a diverse influence of concurrent *D. repens* infection on measures of pathology in babesiosis.

In our study *D. repens* DNA was found only in dogs and in one cat from the area of Warsaw, spanning a radius of about 60 km from the city center in the region of Mazovia (Masovian voivodeship). To our knowledge, this is the first finding of *D. repens* in a cat in central Europe. Interestingly, detection of the filarial endosymbiotic bacterium, *Wolbachia*, proved to be a more sensitive detection technique than the direct detection of *D. repens* DNA, detecting 2 and 1 positive cat, respectively, but this conclusion needs further confirmation due to the very limited number of positive samples in our study.

Mazovia was first recognized as a new endemic region for canine dirofilariosis in Poland [14, 25] and recent studies, including the present one, confirm the stable continuous transmission of parasites in this region [8, 9]. The prevalence of *D. repens* recorded in this study in dogs (38 %) is within the range of previously reported rates in this area (20–60 %) and is typical for endemic regions in much warmer climates [2]. Such a high prevalence in dogs indicates a high risk of emergence of human dirofilariosis in the region, as seroprevalence of *D. repens* in humans may reach the same values as prevalence in dogs in some locations [1, 2]. Interestingly, all the dogs included in this study were ‘healthy’, as were the dogs in previous studies [9, 19], so no directed treatment to control microfilaraemia in these dogs had been implemented, creating a continuous source of infection for the vectors.

We were unable to discover *D. repens* DNA in more than 100 dogs from three other macroregions of Poland. This is generally in agreement with the results of a previous study on 1588 dogs from Poland, reporting a much lower prevalence of *D. repens* in dogs from northern, western and southern Poland, spanning a range of just 0–10 % [10, 21]. Demiaszkiewicz et al. [10] reported higher prevalence in dogs from eastern Poland, ranging between 13–16 % for Podlaskie and Lubelskie voivodeships, in regions not included in the present study. However, the well-established endemic area in central Poland may serve as the source of the spread of the parasite to new regions in Poland, due to translocation of asymptomatic but nevertheless infected dogs.

Central and eastern Poland are also endemic regions for canine babesiosis and we discovered a positive association between infection with these two vector-borne parasites. Prevalence of *D. repens* was 2.5 times higher in dogs with babesiosis than in representatives of a healthy population from the same region and this association was highly significant. Although *D. repens* infections are more prevalent, but generally asymptomatic, in dogs in central Poland, infection with *B. canis* rarely remains asymptomatic [29, 39] although the course of babesiosis may differ from moderate to severe and sometimes even fatal. All the dogs involved in this phase of our study were infected with *B. canis*, presenting a range of symptoms and typically characteristic changes in blood parameters [40–44]. Additionally, to check if there were any asymptomatic co-infections of *B. canis* and *D. repens*, we tested 94 healthy dogs from Mazovia by the same molecular techniques. Despite the high prevalence of *D. repens* (38 %) in these dogs, we detected only one dog from this group as positive for *B.canis* DNA and this individual was free of *D. repens*, so no asymptomatic co-infections were found. However, in 366 microfilaraemic but clinically healthy working dogs in Slovakia, 14 cases of co-infection of *B. canis* and *D. repens* were confirmed [19]. The other explanation can be that the high rate of co-infection between mosquito-borne dirofilariosis and tick-borne babesiosis is the result of generally higher exposure of the dogs to these vectors, i.e. through their spatio-temporal activity patterns or the lack of treatment for ectoparasites in this group of dogs.

The possible mechanisms underlying the impact of *D. repens* on the course of babesiosis are currently unknown. In a recent review on common features in malaria and babesiosis, two principal factors linked to pathogenecity were identified, one associated with immunological stimulation and the second with adhesion and sequestration of infected RBC [45]. Heavy burdens of microfilariae in capillary vessels may interfere with RBC adhesion, disturbing blood flow through vital organs. In our study, analyses of various blood parameters revealed a quite different pattern for parameters associated with blood morphology and anemia and for biochemical parameters. In the first instance, as we might have expected, the most pronounced changes were observed in dogs with co-infection compared with dogs infected only by *B. canis* (lower numbers of thrombocytes, hematocrit, hemoglobin concentration), but of these only the number of thrombocytes was significantly lower. Contrary to our expectation, the biochemical parameters displayed more pronounced changes in dogs infected solely by *B. canis*, implying that these animals were experiencing more severe episodes of babesiosis with hepatic and renal failure. This finding has similarities to the conclusions of a long-term study on malaria and intestinal helminths in humans from Thailand [46, 47]. Observational studies in Thailand have shown that although the incidence of *Plasmodium falciparum* malaria was twice as high in helminth-infected patients, there was a 64 % reduction of cerebral malaria and an 84 % reduction of acute renal failure in patients co-infected with malaria and helminths in comparison to those without helminths [46]. Although fever was lower in patients with co-infections, they suffered from more severe anemia, but generally it was concluded that helminth invasions had a protective effect against severe and fatal course of *P. falciparum* malaria.

The possible mechanisms underlying these phenomena (higher susceptibility to *B. canis* infection but less severe babesiosis) require further investigation. The higher probability of developing babesiosis after *B. canis* infection in *D. repens*-infected dogs, may be the result of a depression of the immune response against pre-erythrocytic stages (sporozoites), as proposed for *P. falciparum* [46, 47]. As helminths, including *D. repens*, are long-lived parasites stimulating a Th-2 type response profile with low production of pro-inflammatory cytokines, they have a marked depressive effect on other immune pathways including the Th-1 type responses that are considered to be host protective in the case of intracellular parasites such as *Babesia* spp. [45]. Suppression of the Th-1 arm of the immune system would inevitably reduce pathology associated with effector mechanisms unleashed during Th-1 driven immune responses [45].

The much lower counts of thrombocytes in dogs with co-infection, may be associated with the recently discovered significant role of these cells in both innate and adaptive immunity against a range of pathogens [48]. Recruitment of platelets in a response targeting *B. canis*-infected RBCs and microfialariae may be the cause of the considerable reduction in their numbers in the circulation of dogs with co-infections. However, although in our study the differences in several blood parameters (i.e. ALP) were significant or showed borderline significance (i.e. for AST, ALT), the group of dogs with the single infection was quite small (*n* = 5) and the output of these analyses should be treated with some degree of caution at this stage.

Our study has several limitations. The sensitivity of PCRs was not determined so prevalence of *D. repens* could be underestimated. The number of sampled dogs outside the Warsaw area is limited. The data on blood parameters was available only for dogs suspected of babesiosis and similar data is missing for dogs infected only with *D. repens*. The main reason for this lack is that dirofilariosis is a relatively new health problem and is very rarely diagnosed and treated in routine vet care of pet dogs. Thus, it was impossible to include a group of samples from dogs with clinical symptoms suspected of suffering only from dirofilariosis. We have data on blood parameters only for two dogs infected solely with *D. repens* (within normal ranges for the majority of parameters as follows: mean RBC = 7.12 T/l; mean leucocytes = 6.15 G/l; mean hemoglobin = 10.8 mmol/l; mean hematocrit = 0.495 l/l; mean thrombocytes = 295.5 G/l; mean AST = 27 U/l; mean ALT = 43 U/l; mean ALP = 37 U/l, mean creatinine = 1.15 mg/dl; mean serum urea = 50.5 mg/dl). Not much conclusions can be drawn from these values and further study is needed to fill this gap.

## Conclusions

In summary, a very high rate of co-infection with *B. canis* and *D. repens* in dogs treated for babesiosis and the lack of asymptomatic co-infections in healthy dogs suggest that microfilaremic dogs are more prone to developing symptomatic babesiosis. However, contrary to our predictions, the values of biochemical parameters in dogs with co-infection were closer to those of healthy dogs than those solely infected with *B. canis* suggesting milder babesiosis in these animals. These findings are important due to the current spread of dirofilariosis and babesiosis in central Europe, which being mostly undiagnosed has resulted in large numbers of untreated microfilaraemic dogs in the region, which may be more prone to babesiosis. The possible protective effect of the nematode infection on hepatic or renal dysfunction in canine babesiosis is intriguing and its mechanisms require further investigations.

## Abbreviations

ALP: Alkaline phosphatase
ALT: Alanine aminotransferase
ANOVA: Analysis of variance
AST: Aspartate aminotransferase
CL: Confidence Interval
DNA: Deoxyribonucleic Acid
EDTA: Ethylene Diamine Tetra-acetic Acid
IBM: International Business Machines
MCHC: Mean Corpuscular Hemoglobin Concentration
MCV: Mean Corpuscular Volume
NS: Not significant
*P*: Probability values
PCR: Polymerase Chain Reaction
RBC: Red Blood Cell
RNA: Ribonucleic acids
SEM: Standard error of the mean
SPSS: Statistical Package for the Social Sciences
